# Does ankyloglossia interfere with breastfeeding in newborns? A cross-sectional study

**Published:** 2021-04-06

**Authors:** Ana Clara Souza-Oliveira, Poliana Valdelice Cruz, Cristiane Baccin Bendo, Wallysson Costa Batista, Maria Cândida Ferrarez Bouzada, Carolina Castro Martins

**Affiliations:** ^1^Dental School, Universidade Federal de Minas Gerais, Belo Horizonte, Brazil; ^2^Medical School, Universidade Federal de Minas Gerais, Belo Horizonte, Brazil

**Keywords:** ankyloglossia, tongue-tie, lingual frenulum, newborn, bottle feeding, oral health, preterm birth

## Abstract

**Background and aim:**

Ankyloglossia is a congenital anomaly that can affect breastfeeding. The aim was to evaluate the prevalence of ankyloglossia in newborns and breastfeeding difficulties reported by mothers; assess possible factors that may interfere with breastfeeding.

**Methods:**

A cross-sectional study was conducted with 391 pairs of mothers/newborns at a university hospital. A pediatric dentist examined the oral cavity of the newborns for the occurrence of ankyloglossia. We analyzed medical records and the mothers answered a self-administered questionnaire to assess birth variables, breastfeeding difficulties, and sociodemographic factors. We calculated prevalence ratios (PRs) of breastfeeding difficulties according to the independent variables.

**Results:**

The mean age of the newborns was 2.5±2.9 days and 52% were male. The prevalence of ankyloglossia was 15% and 91.4% of mothers reported not having breastfeeding difficulties. Ankyloglossia was not associated with breastfeeding difficulties (PR: 0.5; 95% CI: 0.2–1.4). Mothers with a low income (PR: 0.5; 95% CI: 0.3–0.8), those who received instructions on breastfeeding (PR: 0.4; 95% CI: 0.2–0.9), and those who breastfed exclusively (PR: 0.3; 95% CI: 0.1–0.8) had fewer breastfeeding difficulties.

**Conclusion:**

Successful breastfeeding was more dependent on being born at full term, the family income, receiving guidance with regard to breastfeeding, and exclusive breastfeeding. Although ankyloglossia was not associated with breastfeeding, future prospective studies should evaluate the long-term factors that may interfere with breastfeeding.

**Relevance for patients:**

This study brings a new perspective on the importance of assessing ankyloglossia and breastfeeding difficulties, reinforces the benefits of exclusive breastfeeding and the need for breastfeeding instructions, as well as the need to evaluate breastfeeding before making a decision regarding frenectomy.

## 1. Introduction

The lingual frenulum is a fold of mucous membrane that connects from the floor of the mouth to the midline of the lower part of the tongue, helping to stabilize the base of the tongue without impairing its movement [1]. However, a congenital anomaly denominated ankyloglossia, commonly known as a tongue-tie, is a condition in which a small portion of the tongue tissue that should have undergone apoptosis during embryonic development remains attached to the sublingual surface inserted in the anterior portion near the tip of the tongue, restricting its movement [2].

Studies on the prevalence of ankyloglossia have used different diagnostic criteria and different age groups of children. Based on these studies, the prevalence ranges from 1.7% to 12.1% [3,4]

Abnormal tongue movements may interfere with breastfeeding, as newborns with limited tongue mobility may not be able to latch onto the nipple with an adequate seal during breastfeeding, which can result in nipple pain, nipple fissures, and ineffective sucking, predisposing the child to early weaning [5].

Breastfeeding is important to the physical and emotional development of infants, the prevention of diseases, the promotion of immunological health, and even in the prevention of cancer in mothers. It strengthens the bond between mother and child, nourishes and protects the newborn, and is an effective way to reduce child morbidity and mortality [6]. Ankyloglossia may make breastfeeding less efficient and decrease the supply of milk, with negative repercussions for weight gain and growth, leading to the need for an infant formula [7].

There are reports of a possible association between the occurrence of ankyloglossia and breastfeeding difficulties [3,8]. However, the lack of standardized criteria for the determination of ankyloglossia can lead to late diagnosis, which can consequently affect breastfeeding. It is important to diagnose this condition correctly and evaluate how limited tongue movements can affect breastfeeding. Therefore, the aims of the present study were to 1) evaluate the prevalence of ankyloglossia in newborns and breastfeeding difficulties reported by mothers and 2) evaluate possible factors that may interfere with breastfeeding. The null hypothesis is that the prevalence of ankyloglossia and breastfeeding is low and ankyloglossia does not interfere with breastfeeding. The alternative hypothesis is that ankyloglossia is the main factor that interferes with breastfeeding.

## 2. Materials and Methods

### 2.1. Sample characteristics and study design

The present cross-sectional study is part of larger study that included mothers and their newborns at a university hospital [9]. We collected data from September 2016 to April 2017 on newborns of both sexes born at the university hospital of the Federal University of Minas Gerais, Belo Horizonte, Brazil. We excluded newborns with systemic conditions or congenital syndromes and those in the intensive care unit (ICU). This study received approval from the local institutional review board (CAAE #57295316.3.0000.5149) and all mothers signed a statement of informed consent before taking part in the study.

To calculate the sample size, we considered 4.8% incidence of ankyloglossia in newborns based on the study by Messner *et al*. (2000) [10], a margin of error of 3.0%, and 99.0% confidence interval. The minimum sample size was 337, to which we added 20.0% to compensate for possible losses, reaching a final sample of 404 newborns.

### 2.2. Calibration process

A dentist performed the clinical examination of the newborns for ankyloglossia. The examiner had undergone training and calibration exercises under the supervision of an experienced pediatric dentist. Training consisted of the discussion of photographs showing cases and non-cases of ankyloglossia. Intraexaminer (Kappa=0.9) and interexaminer (between the examiner and experienced pediatric dentist) (Kappa=0.9) agreement was adequate (Kappa > 0.80) [11] and the examiner was considered able to conduct the main study.

We conducted a pilot study to test the methods. The questionnaire was applied to 10 mothers. The examiner assessed newborns for ankyloglossia observing the position of the lips at rest and the positioning of the tongue during crying. The lateral margins of the tongue were raised with the right and left index fingers and the examiner observed whether it was possible to view the frenulum. Thickness and attachment to the tongue and the floor of the mouth were assessed when the frenulum was visible [12]. The results of the pilot study revealed no need for changes to the proposed methods

### 2.3. Data collection

The examiner evaluated the newborns for ankyloglossia in their cribs using disposable cotton swabs, a headlamp, and personal protective equipment (white coat, disposable gloves, mask, head cap, and protective eyewear). A research assistant took notes during the clinical diagnosis. Ankyloglossia was recorded based on the Neonatal Tongue Screening Test of the Lingual Frenulum Protocol for Infants developed and validated by Martinelli et al. (2016) [13]. Initially, the position of the lips at rest was assessed while the newborn was asleep. Next, with the newborn awake, the attachment of the frenulum to the tongue and floor of the mouth was examined and the thickness of the frenulum was categorized (thin or thick). The positioning of the tongue during crying was also analyzed; the shape of the tongue apex when elevated could be round with a slight V-shaped or heart-shaped slit, revealing the connection of the frenulum to the floor of the mouth [14].

Data on the sex of the newborn, gestational age, and birth weight were collected from the medical records. Preterm birth was considered any birth <37 weeks and full term was any birth ≥37 weeks. Birth weight was categorized as low (LBW <2500 g) or normal (NBW ≥2500 g) [15].

Each mother answered a self-administered questionnaire addressing her age, whether she intended to offer a pacifier to the newborn, whether she had received instructions on breastfeeding, whether she could breastfeed exclusively, and whether she had experienced breastfeeding difficulties. The questionnaire also addressed information on family income using the Brazilian minimum monthly wage (BMMW=U$ 288 at the time of data collection) as the unit of reference. Socioeconomic level was categorized as ≤ twice the BMMW (considered “low”) and > twice the BMMW (considered “high”) [9].

### 2.4. Statistical analysis

We performed data analysis with the aid of the Statistical Package for the Social Sciences (SPSS, IBM Corp. for Windows, version 20.0, Armonk, NY). We descriptively analyzed the categorical variables (frequency distribution) and continuous variables (mean and standard deviation). The main variable was breastfeeding difficulties reported by the mother on the questionnaire (yes or no). The other variables were mother’s age, sex of the newborn, gestational age (preterm or full term), birth weight (LBW or NBW), ankyloglossia (yes or no), whether the mother intended to offer a pacifier (yes or no), whether the mother had received instructions to breastfeed (yes or no), whether the mother could breastfeed exclusively (yes or no), and income (high or low).

We used a Poisson regression model with robust variance to test associations between breastfeeding difficulties with the other variables. We ran a multivariate Poisson regression model to control for possible confounders. Variables with *P*≤0.20 in the bivariate analysis were incorporated into multivariate model and those with *P*<0.05 remained in the final model (significance level: 5%).

## 3. Results

We included 391 pairs of mothers/newborns (52% of the newborns were male). Newborn age ranged from 0 to 27 days of life (mean: 2.5±2.9 days). Birth weight ranged from 1690 to 4700 grams (mean: 3.0±514.5). Gestational age ranged from 33 to 42 weeks (mean: 38.3±1.7). The mothers had a mean age of 27.4±7.2 years. About 86% of mothers (n=336) reported not having breastfeeding difficulties. The prevalence of ankyloglossia was 15% (n=58).

Among the 58 mothers of newborns with ankyloglossia, 53 (91.4%) reported no breastfeeding difficulties. Among those of newborns without ankyloglossia (n=332), 85.2% (n=283) reported no breastfeeding difficulties (*P*=0.229). In the bivariate analysis, preterm birth (0.0029), absence of instruction to breastfeed (*P*=0.039), lack of exclusive breastfeeding (*P*=0.022), and high income (*P*=0.013) were associated with difficulties breastfeeding (Table 1).

**Table 1:** Bivariate and multivariate analyses between breastfeeding difficulties and other variables in newborns

Breastfeeding difficulties			Bivariate model unadjusted		Multivariable model adjusted	
		
Variables	No	Yes	PR (95% CI)	*P*-value	PR (95% CI)	*P*-value
Newborn’s sex						
Male	175 (87.1)	26 (12.9)	1		-	-
Female	161 (85.2)	28 (14.8)	1.1 (0.6–1.8)	0.592	-	
Mother’s age						
Up to 19 years	50 (86.2)	8 (13.8)	1			
20 to 35 years	232 (84.7)	42 (15.3)	1.1 (0.5–2.2)	0.768	-	-
36 years or older	51 (94.4)	3 (5.6)	1.8 (0.1–1.4)	0.162	-	-
Gestational age						
Full term	281 (87.5)	40 (12.5)	1		1	
Preterm	44 (77.2)	13 (22.8)	1.8 (1.04–3.2)	0.034	1.8 (1.1–3.2)	0.029
Birth weight						
Normal weight	289 (86.5)	45 (13.5)	1		-	-
Low weight	45 (84.9)	8 (15.1)	1.1 (0.5–2.2)	0.748	-	
Ankyloglossia						
No	283 (85.2)	49 (14.8)	1		-	-
Yes	53 (91.4)	5 (8.6)	0.5 (0.2–1.4)	0.229	-	
Intention to use pacifier						
No	224 (87.5)	32 (12.5)	1		-	-
Yes	108 (84.4)	20 (15.6)	1.2 (0.7–2.0)	0.397	-	
Breastfeeding instructions						
No	17 (70.8)	7 (29.2)	1		1	
Yes	316 (87.1)	47 (12.9)	0.4 (0.2–0.8)	0.019	0.4 (0.2–0.9)	0.039
Exclusive breastfeeding						
No	6 (60.0)	4 (40.0)	1		1	
Yes	329 (86.8)	50 (13.2)	0.3 (0.1–0.7)	0.007	0.3 (0.1–0.8)	0.022
Income						
High	78 (80.4)	19 (19.6)	1		1	
Low	257 (88.0)	35 (12.0)	2.0 (1.1–3.3)	0.036	0.5 (0.3–0.8)	0.013

Pearson’s Chi-squared test; b Fisher’s exact test. Results in bold type are statistically significant at 5% level. Poisson regression model with robust variance for multivariate analyses. Multivariable model: All variables with *P<*0.20 in bivariate analyses incorporated into model

In the final multivariable model, the following variables were associated with a lower prevalence ratio (PR) of breastfeeding difficulties: Mothers having received breastfeeding instructions (PR: 0.4; 95% CI: 0.2–0.9), exclusive breastfeeding (PR: 0.3; 95% CI: 0.1–0.8), and low income (PR: 0.5; 95% CI: 0.3–0.8). The prevalence of breastfeeding difficulties was higher among preterm infants (PR: 1.8; 95% CI: 1.1–3.2). Ankyloglossia was not associated with breastfeeding difficulties (PR: 0.5; 95% CI: 0.2–1.4) (Table 1).

## 4. Discussion

Contrary to what was expected, the alterative hypothesis was rejected, as ankyloglossia was not a factor for breastfeeding difficulties in this study. Moreover, mothers who had received breastfeeding instructions, those who breastfed exclusively, and those with a low income had fewer breastfeeding difficulties, whereas mothers of preterm newborns experienced more breastfeeding difficulties.

Conflicting data are found in the literature on the association between ankyloglossia and breastfeeding difficulties. One study reported that ankyloglossia was responsible for 12.8% of serious breastfeeding problems [16]. Another study reported that 83% of mothers with newborns affected by ankyloglossia breastfed successfully (similar to the finding in the present investigation), confirming that ankyloglossia alone rarely causes breastfeeding problems [10]. A systematic review reported that not all babies with ankyloglossia have breastfeeding problems and many adapt to this condition [17]. The rationale for the breastfeeding difficulties resulting from ankyloglossia is that abnormal, restricted tongue movements can cause persistent nipple pain, bleeding, cracked/ulcerated nipples, and mastitis in mothers whose infants have this condition [10,18].

Different diagnostic methods are used to evaluate breastfeeding difficulties, which may explain the controversy regarding the treatment of ankyloglossia [10]. The Hazelbaker Assessment Tool for Lingual Frenulum Function (HATLFF) can be used for the objective determination of the degree of ankyloglossia, but is based on subjective clinical assessments and does not address issues related to breastfeeding difficulties [16,19]. Moreover, although the HATLFF tool is validated, there are concerns regarding its reliability [20]. The Bristol Tongue Assessment Tool (BTAT) provides an objective, simple assessment of the severity of tongue attachment [21]. The tongue-tie and breastfed baby (TABBY) assessment tool was proposed to improve the diagnosis and has items related to the position of the frenulum, tongue shape, lifting movements (tongue on the palate), and expulsion (tongue out of the mouth) [22]. However, all tools are limited with regard to the assessment of feeding [22]. Due to the absence of protocols for the simultaneous assessment of characteristics of the lingual frenulum and the functions of sucking and swallowing during breastfeeding, the Martinelli Protocol was chosen [12] for the present study.

However, the diagnosis used in our study is limited due to the fact that we only considered the modified diagnosis proposed by Martinelli et al. (2012) [12] and only the anatomical definition was considered; we did not take into account the functional definition. The prevalence of ankyloglossia varies widely in the literature. Haham et al. (2014) found that 99.5% of newborns had a visible sublingual frenulum [3]. Maya-Enero et al. (2020) stated that virtually all children have a lingual frenulum to some degree, but the authors found that only 3.5% had symptomatic tongue-tie requiring treatment or surgical intervention [23]. The prevalence of ankyloglossia in the present study was 15% considering modified criteria proposed by Martinelli et al. (2012) [12].

It seems that the diagnosis of ankyloglossia has improved in the last decade, which has contributed to an increase in frenectomy procedures. Walsh et al. (2017) demonstrated that the incidence of children diagnosed with ankyloglossia increased more than 8-fold between 1997 and 2012, while the incidence of children undergoing the frenectomy procedure increased more than 9-fold in the same period [24].

A study reported that frenectomy for infants with ankyloglossia can improve breastfeeding and relieve nipple pain [18]. Another study also found a significant reduction in nipple pain after frenectomy, but not enough to improve breastfeeding [25]. The evidence that frenectomy improves breastfeeding efficiency and speech development is insufficient and questionable [26,27]. In the present study, no newborn underwent frenectomy during hospitalization. In our view, the impact of ankyloglossia on breastfeeding difficulties should be carefully evaluated before planning frenectomy soon after delivery.

Mothers of preterm newborns had more breastfeeding difficulties in the present study, which was expected, as premature newborns have weak suction and low muscle tone, which can decrease the volume of milk obtained. Moreover, these newborns can have uncoordinated oral movements, which is related to a reduction in the intake of nutrients as well as a greater risk of dehydration and insufficient caloric intake [28].

Premature babies are at a disadvantage in terms of feeding skills. They are born with low-energy reserves and require high-energy intake. As their feeding skills may be compromised due to their premature development, meeting the nutritional and hydration needs of these infants poses a challenge to healthcare providers and these children are more likely to develop breastfeeding difficulties [29,30]. Moreover, considering the myriad emotions involved in premature delivery, pumping and maintaining the milk supply can be difficult for mothers when they do not receive instructions or support for breastfeeding. Studies have shown that breastfeeding guidelines have a positive effect on breastfeeding success and are an additional tool for promoting breastfeeding in preterm newborns [31].

We strongly support offering breastfeeding instructions and assistance to mothers after delivery. The present findings are in agreement with data from a trial conducted with mothers who received breastfeeding instructions by telephone and daily support from nurses during hospitalization and home visits for 3 weeks. The group of mothers that received this assistance spent an average of 40 more hours breastfeeding their infants and used infant formulas significantly less than those who did not receive this assistance. There was also less of a need for visits to the health-care provider as well as fewer tests and medical appointments [32]. Breastfeeding involves more than a mother’s desire to breastfeed. It is a dynamic process affected by socioenvironmental factors related to experiences after the onset of breastfeeding [33]. One study showed that more than half of mothers discontinued breastfeeding due to the perception of inadequate milk or other breastfeeding problems and consequently began using infant formulas [34]. The discontinuation of breastfeeding may be related to depressive symptoms or the need to return to work or school. Breastfeeding instructions should focus on nutritional counseling and breastfeeding should be supported both during postpartum hospitalization and immediately after discharge [35].

Breastfeeding instructions can indeed improve breastfeeding. A previous study showed that educational breastfeeding programs influenced mothers to perform exclusive breastfeeding for 6 months [36]. Knowledge on the benefits of breastfeeding (disease prevention and benefits for the newborn’s immune system) can influence a mother’s decision to insist on exclusive breastfeeding. Moreover, exclusive breastfeeding rather than bottle feeding prevents contamination by water, bottles, and unsterile utensils [37]. Exclusive breastfeeding for 6 months and continued through the 1^st^ year of life can prevent infant mortality for up to 5 years [38]. There is evidence that a mother’s intentions are related to the period of breastfeeding, as a high correlation was found between a mother’s intention to breastfeed exclusively for 6 months and the duration of exclusive breastfeeding [39].

Breastfeeding difficulties may be associated with socioeconomic indicators, such as income, educational level, and ethnicity. Individuals with a lower socioeconomic status, those with a lower educational level, and minority groups may be less likely to breastfeed [40]. One study found a dose–response relationship between the prevalence of breastfeeding and educational level. The prevalence of breastfeeding was 65% among mothers with a middle school education (PR: 1.1; 95% CI: 1.0–1.1), 71% among those with a high school/vocational school education (PR: 1.1; 95% CI: 1.0–1.2), and 77% among those with a college education or higher (PR: 1.2; 95% CI: 1.1–1.2) [41]. In contrast, mothers with a lower income were more likely to breastfeed in the present investigation, which is in agreement with data described in a previous study [42]. Mothers from minority groups were more likely to breastfeed compared to American mothers with a higher income level, whereas the latter group was more likely to offer infant formula to their children [42]. Mothers with a lower income may have less access to infant formula, which may encourage breastfeeding as a cheaper source of nutrition for their children compared to infant formulas.

Our study demonstrates that, in addition to promoting breastfeeding, it is necessary to provide instructions for breastfeeding and public health policies are needed to encourage this practice. Mothers need to be instructed on how to achieve successful breastfeeding and need to be aware of the benefits for their infants, such as reductions in the occurrence of diarrhea, gastrointestinal infections, and atopic eczema [43].

This study has limitations that should be considered. The sample was from a single hospital and no follow-up of the newborns was performed to find out whether breastfeeding remained successful in the long term. The use of modified criteria proposed by Martinelli et al. (2012) [12] with only the anatomical definition is a limitation, since breastfeeding was not assessed directly. This also results in a limitation in the prevalence of ankyloglossia and affects the prevalence of breastfeeding difficulties. However, this study offers a new perspective on the importance of assessing ankyloglossia and breastfeeding difficulties, addressing the importance of exclusive breastfeeding and breastfeeding instructions, as well as the need to evaluate breastfeeding before making a decision regarding frenectomy.

## 5. Conclusion

Most of the newborns with ankyloglossia were able to breastfeed and guidelines for breastfeeding are directly linked to the ease of breastfeeding. Mothers who could breastfeed exclusively and those with a lower income had fewer breastfeeding difficulties. Although our results show that ankyloglossia did not affect breastfeeding, there is a need for prospective studies with a long-term evaluation of newborns to determine possible factors associated with the interruption of breastfeeding.
